# Effect of ezetimibe add-on therapy over 52 weeks extension analysis of prospective randomized trial (RESEARCH study) in type 2 diabetes subjects

**DOI:** 10.1186/s12944-017-0508-4

**Published:** 2017-06-24

**Authors:** Kentaro Sakamoto, Mitsunobu Kawamura, Takayuki Watanabe, Keiko Ashidate, Takahide Kohro, Akira Tanaka, Yasumichi Mori, Motoki Tagami, Tsutomu Hirano, Tsutomu Yamazaki, Teruo Shiba, RESEARCH Study Group

**Affiliations:** 1 Department of Diabetes and Metabolism, Toho University Ohashi Medical Center, 2-17-6, Ohashi, Meguro-ku, Tokyo, 1538515 Japan; 2 Division of Endocrinology and Metabolism Department of Internal Medicine, Tokyo Teishin Hospital, Tokyo, Japan; 3 Department of Internal Medicine, Yokohama City Minato Red Cross Hospital, Tokyo, Kanagawa Japan; 4 Department of Internal Medicine, Kudanzaka Hospital, Tokyo, Japan; 5 Department of Medical Informatics / Cardiology, Jichi Medical University, Tochigi, Japan; 6 Nutrition Clinic, Kagawa Nutrition University, Tokyo, Japan; 7 Department of Endocrinology and Metabolism, Toranomon Hospital, Tokyo, Japan; 8 Sanraku Hospital, Life-style related Disease Clinic, Tokyo, Japan; 9 Department of Medicine Division of Diabetes Metabolism and Endocrinology, Showa University School of Medicine, Tokyo, Japan; 10 Clinical Research Support Center, The University of Tokyo Hospital, Tokyo, Japan; 11 Division of Diabetes and Metabolism, Mitsui Memorial Hospital, Tokyo, Japan

## Abstract

**Background:**

Lowering cholesterol levels decreases the risk of atherosclerotic diseases. Effective ways to stably reduce LDL-C level are warranted in type 2 diabetic patients, a high-risk population for CVD, with various anti-diabetic therapeutic background. The RESEARCH study focuses on LDL-C reduction in this population along with modifications of the lipid profiles. We evaluated long-term ezetimibe add-on therapy in T2DM patients with hypercholesterolemia.

**Methods:**

In a randomized, multicenter, open-label, prospective study, a total of 109 T2DM patients not attaining LDL-C target value despite first-line dose statin (10 mg of atorvastatin or 1 mg of pitavastatin) therapy in Japan were recruited. We investigated the difference in cholesterol lowering effect between ezetimibe (10 mg) add-on statin (EAT) group and double-dose statin (DST) group. Changes of parameters related to atherosclerotic event risks were assessed.

**Results:**

The reduction of LDL-C was larger in the EAT group (28.3%) than in the DST group (9.2%) at 52 weeks as well as the primary endpoint of 12 weeks. EAT achieved significant lower levels of TC and apo B, respectively. Both treatments attained significant reduction in sd-LDL-C or hsCRP on this long-term basis. Notably, sd-LDL-C in EAT reduced as low as 36.1 ± 14.9 mg/dl to reach near the threshold (35.0 mg/dl) for atherosclerosis with significantly higher achievement rate (55.6%) than DST treatment. Simultaneously, hsCRP reduction by EAT attained as low value as 0.52 ± 0.43 mg/l.

**Conclusions:**

In the present 52-week long-term period, ezetimibe add-on therapy showed a robust advantage in lowering LDL-C and in attaining target LDL-C values compared with the doubling of statin dose. Moreover, it’s meaningful that sd-LDL, powerfully atherogenic lipoprotein, exhibited prominent decrease consistently prominently by ezetimibe add-on therapy. DM patients with hypercholesterolemia are at high risk for CAD, and adding ezetimibe onto usual-dose statin treatment in Japan has been suggested as the first-line therapy for those DM patients who failed to attain the target LDL-C value (UMIN000002593).

## Background

Cardiovascular disease remains the leading cause of morbidity and mortality for patients with type 2 diabetes in spite of the multi-factorial interventions recently introduced to control CVD risk factors [1, 2]. One of the primary causes of this high morbidity and mortality is the poor prognosis of hypercholesterolemia associated with atherogenic dyslipidemia linked to triglyceride-rich lipoproteins. Lipid reduction by 3-hydroxy-3-methyl-glutaryl-CoA reductase inhibitor (statin) therapy also plays an essential therapeutic role for type 2 diabetes, but it still fails to frequently attain the target LDL-C level. Ezetimibe is a relatively new, non-statin cholesterol-lowering agent that inhibits the absorption of dietary and biliary cholesterol across the intestinal wall by suppressing NPC1L1 protein. Ezetimibe thus complements the effects of statins, which inhibit cholesterol synthesis in the liver. The addition of ezetimibe to standard therapy with statin inhibits two sources of cholesterol, which improves the profiles of triglyceride-rich lipoproteins in type 2 diabetes and reduces LDL-C to a degree unattainable by either agent alone.

Despite the aggressive statin therapy now available, a significant proportion of patients still experience cardiovascular events. Until recently, the addition of a nonstatin lipid-modifying agent to statin therapy has conferred no significant increment in cardiovascular benefit over that seen with a statin alone. The combination of fenofibrate and simvastatin fails to reduce the rate of fatal or non-fatal cardiovascular events compared with simvastatin alone, though it does offer possible benefits for type 2 diabetics with both high baseline triglyceride and low baseline HDL-C [3]. Extended-release niacin offers no beneficial effects on CV events and is reported to trigger serious adverse events in subjects with diabetes and to increase the risks of the newly onset DM [4, 5]. IMPROVE-IT is the first clinical outcomes trial to show that a nonstatin LDL-C–lowering agent (ezetimibe) plus statin therapy reduces CVD in patients at a high risk of recurrent CVD events [6]. In a population of high-risk Japanese patients, the combination of atorvastatin and ezetimibe reportedly showed a greater coronary plaque regression than atorvastatin monotherapy, along with significant effects in lowering LDL-C [7]. The efficacy of ezetimibe add-on therapy with statins was thus demonstrated to protect against atherosclerotic cardiovascular disease in a clinical setting via its anti-hyperlipidemic effects.

In a previous report for the primary endpoint of the RESEARCH trial, ezetimibe add-on with high-potency statin was superior to a double dose of basal statin in type 2 diabetic subjects in whom various treatments had failed bring about the target LDL-C goal for the Japanese. This paper describes a 52-week extension of this study undertaken to evaluate the long-term efficacy, safety, and tolerability of ezetimibe add-on with statin for type-2 diabetic patients.

## Methods

### Randomized trial

**Fig. 1:**
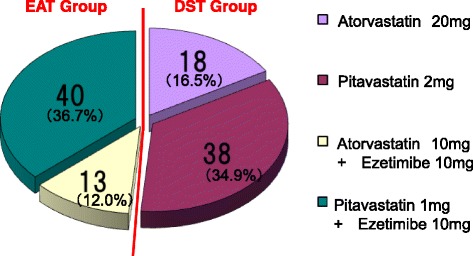
Baseline characteristics of treatment groups. Number of cases in each treatment (total: *n* = 109). Baseline breakdown of statins for each treatment group was shown

RESEARCH (Recognized Effect of Statin and Ezetimibe therapy for Achieving the LDL-C goal) was conducted as a randomized, doctor-oriented, multicenter trial to compare the effects of double-dose statin versus ezetimibe-plus-statin on the serum LDL-C concentration of type-2 diabetes patients with various anti-diabetic therapeutic backgrounds. The trial was initially reported elsewhere [8], and the baseline demographic characteristics were also shown. Baseline statin was 10 mg of atorvastatin or 1 mg of pitavastatin. 78 subjects received pitavastatin (38 for the DST group and 40 for the EAT group), and 31 received atorvastatin (18 for the DST group and 13 for the EAT group). (Fig.1)

The patients consented to participate in an extended treatment period from the initial 12 weeks to 52 weeks and were enrolled in the analysis. Interval data were also sampled at 26 weeks. Intention To Treat (ITT) and Per Protocol analyses were performed on a subgroup of 5 subjects (2 from the EAT group and 3 from the DST group) by administering an intensified lipid-lowering therapy for failure to achieve the target LDL-C values. The ITT analysis was modified by excluding any subject with incomplete data for any reason.

### Patients

Of the recruited subjects, all 109 of the patients enrolled at 12 weeks agreed to take part in the extended analysis. Agents for diabetes treatment were also shown in the previous report [8]. Seven patients, however, were forced to drop out from the extension study because of adverse events. Eighty-five patients underwent sampling at 52 weeks.

### Randomization and study treatment

As mentioned in a previous report, the patients had been allocated to two groups by dynamic allocation methods, one for the ezetimibe-add-on therapy (the EAT group) and the other for statin therapy at double dose (the DST group). When the LDL-C targets had not been reached after 12 weeks of treatment and the patients and their doctors agreed, the patients in the EAT group began higher doses of statin and the patients in the DST group began higher doses of either statin or ezetimibe. The initial assignment was sustained according to the ITT policy for the analysis. The patients received none of the following agents during the study: statins other than atorvastatin or pitavastatin, anion-exchanging resin agents, fibrates, nicotinic acids, eicosapentaenoic acid, probucol, or other lipid-lowering agents.

### Outcome measures

All of the primary and key secondary efficacy variables were pre-specified for the analysis at 12 weeks. In the extension study these variables were basically re-evaluated to assess how well the efficacy was sustained. The long-term safety and tolerability of both treatments over 52 weeks were assessed by collecting data on adverse events, laboratory parameters, and vital signs. The efficacy outcome was evaluated based on the ITT policy. The initially defined primary endpoint at 12 weeks was the percent change in LDL-C from baseline. This point remained our main assessment target for the duration of the study. Apart from the basic lipid parameters of total cholesterol (TC), triglyceride (TG), high-density lipoprotein cholesterol (HDL-C), and apolipoproteins, the re-specified secondary endpoints included the rates at which the target LDL-C values recommended by the guidelines [9] were achieved and the values and percent changes in high-sensitivity C-reactive protein (HsCRP), sd-LDL, and remnant-like particle cholesterol (RLP-C). HsCRP was adopted only for the subjects without overt causes of systemic inflammation. Sd-LDL was measured at Showa University (Tokyo, Japan) by the precipitation method described earlier [10]. The CVs for within-run precision were <1.1%, and total CVs were <2.2%. HsCRP (total CVs 2.94%) and RLP-C (total CVs 2.88%) were measured by SRL, Japan. Tolerability was assessed based on general parameters such as AST, ALT, creatinine, and CPK, along with plasma glucose and HbA1c values monitored to detect any worsening of diabetes. Serum insulin level was also examined.

### Statistical analysis

The effects of therapy modification were observed in patients who underwent treatment changes during the extended period after failing to reach the target LDL-C level at 12 weeks. The long-term effects of the regimen in each group were evaluated in the subjects who underwent no treatment modification during the extended period. An ITT analysis and Per Protocol analysis were performed.

In the assessments for the end points, differences between the two groups in categorical variables were evaluated using the chi-square test and differences in continuous variables were evaluated using the Wilcoxon test. The Wilcoxon signed-rank test was used to assess the differences in parameters before and after treatment within each group, excluding the age parameter. A *p* value of less than 0.05 (2-sided) was assumed to indicate significance in all analyses. All data were analyzed using Stata version 12.1 (StataCorp LP, Texas, U.S.A.).

## Results

Of the 109 patients (53 in the EAT group and 56 in the DST group) analyzed at 12 weeks, the same patients in each group were compared at 52 weeks according to the ITT policy and 104 patients (51 in EAT and 53 in DST) were committed for the Per Protocol analysis. The baseline demographics of the patients have already been described in the earlier paper reporting the primary results of the trial. Briefly, no significant differences were found between the groups in coronary risks or laboratory data, while slight but significant differences were observed in the levels of non-HDL-C, apo-B, and sd-LDL. Among the patients who failed to achieve the LDL-C goal at 12 weeks, only 5 (2 in EAT and 3 in DST) received additional drugs permissible under the study protocol and based on the judgments of their doctors.

**Fig. 2:**
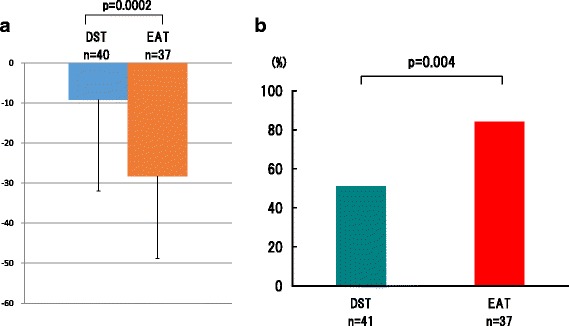
The changes in LDL cholesterol. **a** The percentage reduction from baseline in LDL-C after 52 weeks of treatment: Blue bar represents percent change in DST and orange bar represents percent change in EST. Error bars represent standard deviations. The *p*-value indicates statistically significant difference between groups by Wilcoxon tests. **b** Achievement rates in each treatment group. The bars of DST group are moss green, and EAT group red. The *p*-value indicates statistically significant difference between groups by a chi-square test

**Fig. 3:**
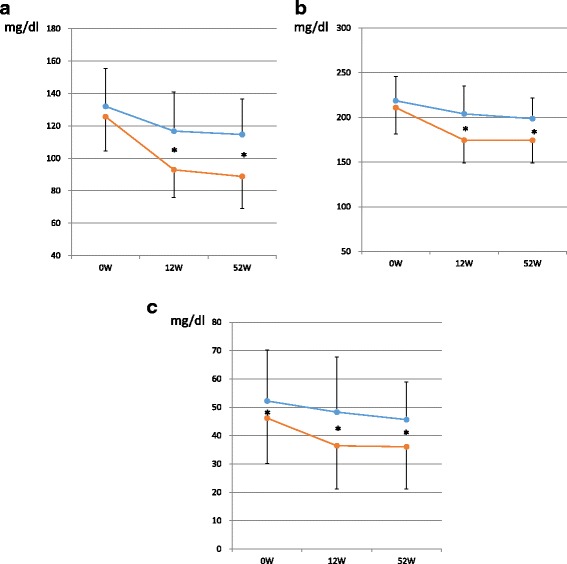
Longitudinal changes in cholesterol values. **a** Longitudinal LDL-C values before and after 12, and 52 weeks of treatments: Blue lines indicates DST therapy while orange lines indicate DST therapy. Error bars represent standard deviations. Asterisks indicate statistically significant difference between groups by Wilcoxon tests. **b** Longitudinal TC values before and after 12, and 52 weeks of treatments: Blue lines indicates DST therapy while orange lines indicate DST therapy. Error bars represent standard deviations. Asterisks indicate statistically significant difference between groups by Wilcoxon tests. **c** Longitudinal sdLDL-C values before and after 12, and 52 weeks of treatments: Blue lines indicates DST therapy while orange lines indicate DST therapy. Error bars represent standard deviations. Asterisks indicate statistically significant difference between groups by Wilcoxon tests

As a primary endpoint of this study, the mean rate of LDL-C reduction at 12 weeks was 24.6% in EAT Group and 10.9% in the DST Group. This larger reduction in the percent change of LDL-C by the add-on ezetimibe was sustained for 52 weeks: 28.3 ± 20.5% in EAT versus 9.19 ± 20.5% in DST Group (*p* = 0.0002) (Fig. 2A). The LDL-C values obtained were 88.8 ± 19.7 mg/dl in EAT Group and 114.7 ± 21.8 mg/dl in DST (comparable to the Japanese target LDL-C level of <120 mg/dl for primary prevention or <100 mg/dl for secondary prevention), which meant that the sufficient reduction conferred by the ezetimibe add-on therapy was sustained (Fig. 3A). The achievement rate for the optimized goal was 82.9% in EAT versus 53.5% in DST (*p* = 0.004) at 52 weeks, which was consistent with the 12-week rates of 89.3% in EAT versus 51.0% in DST. (Fig. 2B)

As to TC, the reduction rate was 16.7 ± 14.1% in the EAT group and 7.6 ± 14.7% in the DST group, respectively. The percent reduction was larger in the EAT group than in the DST group. The absolute value of TC at 52 weeks was 174.3 ± 25.2 mg/dl in EAT versus 198.5 ± 23.3 mg/dl in DST, respectively (Fig. 3B).

TG exhibited a statistically significant increase at 52 weeks from the baseline, and HDL-C significant decrease at 52 weeks from the baseline in EAT group, although the values of both TG and HDL-C (153.2 ± 73.8 mg/dl for TG and 53.7 ± 12.0 mg/dl for HDL-C) showed no significant difference from those of DST group (160.2 ± 73.9 mg/dl for TG and 52.3 ± 8.2 mg/dl for HDL-C) at 52 weeks.

Comparable to the changes in TG and HDL-C values, Apo AI showed no statistically significant differences during the observation period (as 141.7 ± 16.9 mg/dl in EAT group, and 143.7 ± 15.1 mg/dl in DST group at 52 weeks), and Apo E showed no significant reduction at 52 weeks (data not shown).

### Adverse events

Three cases of adverse events were reported before 12 weeks in the EAT group: a common cold in 1 patient, hepatocellular carcinoma in 1, and eczema in 1. The patients with hepatocellular carcinoma and eczema withdrew their consent and were accordingly excluded from the extension study. Adverse events then appeared in another 5 subjects during the extended period: adenocarcinoma of unknown origin in 1 subject of EAT group, lung cancer in 1 of DST group, myelodysplastic syndrome in 1 of DST group, idiopathic thrombocytopenic purpura in 1 of DST group, and ileus by abdominal abscess in 1 of EAT group.

As it is reported that mutations in HMG-CoA reductase and NPC1L1 result in glycemic change [11, 12], worsening in glycemic control was checked as an adverse outcome. HbA1c showed no significant change after both 12 and 52 weeks of intervention (basal: DST 7.27 ± 0.95%, EAT 7.25 ± 0.64%, 52wks: DST 7.22 ± 0.96%, EAT 7.38 ± 0.73%).

As to hepatic or renal function, we observed no clinically meaningful episodes suggestive of worsening of each function or parameter changes defined by the protocol. However, slight but significant elevations of AST and ALT values from the baseline were pointed out in the EAT group within the normal ranges as average (AST: 24.1 ± 11.3 IU/l from 21.3 ± 7.8 IU/l, and ALT: 26.9 ± 18.0 IU/l from 23.5 ± 12.4 IU/l). Regarding renal function, mean serum creatinine level exhibited increases in both groups (0.91 ± 0.34 mg/dl from 0.83 ± 0.28 mg/dl in DST group and 0.82 ± 0.34 mg/dl from 0.76 ± 0.24 mg/dl in EAT group) and the increase in DST group reached statistical significance. No changes were observed in muscle symptoms or the CPK level.

Overall, from the point of clinical significance, the two treatments exhibited similar tolerability and safety profiles.

### Specific parameters of interest

We evaluated other lipid profiles related to insulin resistance and micro-inflammation such as apoB, small dense LDL, RLP-C, and high-sensitivity CRP (hsCRP). HOMA-IR was derived from glucose and insulin values when defined.

ApoB has been reported as a stronger predictor of atherosclerotic events. Apo B was reduced in parallel with LDL-C in both groups at 52 weeks (101.7 ± 17.7 mg/dl in DST group and 83.0 ± 16.6 mg/dl in EAT group) from the baseline (112.5 ± 18.2 mg/dl in DST group and 101.2 ± 14.8 mg/dl in EAT group). The mean reduction rate was significantly higher in EAT group (16.7%) than in DST group (8.2%). ANCOVA denied the confounding of the difference in the baseline values between both groups (data not shown).

Small dense LDL is also known to drive strong atherogenic effects. We have observed superior effects of ezetimibe add-on therapy on small dense LDL at 12 weeks. At 52 weeks, superior effects of the ezetimibe add-on therapy on sd-LDL were sustained. Mean sd-LDL level remained near the threshold (35 mg/dl) of Japanese population for progressive atherosclerosis in EAT group. This sd-LDL outcome (36.1 ± 14.9 mg/dl at 52 weeks) was significantly lower than those in the DST Group (45.6 ± 13.4 mg/dl at 52wk). The sd-LDL-C level at 52wks were significantly different (*p* = 0.0035). (Fig. 3C)

In contrast, remnant lipoprotein particle cholesterol (RLP-C) exhibited no significant changes from baseline in both treatment groups at 52 weeks although a significant reduction in RLP-C was observed at 12 weeks in EAT group (data not shown). These behaviors were accompanied by neither changes in HOMA-IR from the baseline nor differences of HOMA-IR between two groups, suggesting the causes other than insulin resistance itself.

High-sensitivity C-reactive Protein (hsCRP), a marker of micro-inflammation, was also assessed. The hsCRP value was 0.52 ± 0.43 mg/l in EAT group and 0.79 ± 1.30 mg/l in DST group at 52 weeks, respectively. Those who attained the cut-off point of hsCRP in Japan (1.0 mg/l) reached 76.2% in DST group, and 85.7% in EAT group, respectively.

The per protocol analysis generally showed similar results (data not shown).

## Discussion

The add-on regimen of ezetimibe plus baseline statin in this RESEARCH extension trial attained better results than the incremental strategy of baseline statin in improving the atherogenic lipid profile of diabetics. A larger percent reduction of LDL-C at 12 weeks of treatment was presented as the primary endpoint for the RESEARCH trial [13], and the add-on regimen of ezetimibe was superior to the incremental strategy of baseline statin during the extended treatment period of 52 weeks in terms of both the percentage reduction and absolute value of LDL-C. The magnitude of the LDL-C reduction was maintained in both treatment groups in percent terms and as an absolute mean value over the 52 weeks. The rate of LDL-C target achievement was stable in both treatment arms after 12 weeks, and an increase of about 30% in the achievement rate was maintained by the ezetimibe add-on therapy over the extended period of 52 weeks. The improvement in total cholesterol was also sustained during the 52 weeks of treatment in percentage terms and as an absolute value. The superior effect of ezetimibe in this trial was clinically meaningful, similar to the results reported from earlier trials of shorter duration [14, 15], and similar to another extension trial of 48 weeks [16] in which ezetimibe was added to statin therapy for primary hyper-cholesterolemic patients irrespective of co-morbidity with diabetes. In comparison with a high-intensity treatment with atorvastatin 80 mg, the co-administration of ezetimibe with simvastatin has been shown to be more effective in reducing LDL-C and in achieving target LDL-C goals [17, 18]. In subjects with type-2 diabetes or metabolic syndrome, ezetimibe (10 mg) with simvastatin (20 mg) at the recommended starting dose for Caucasian was superior to 10 mg/day of atorvastatin at the end of 6 weeks of treatment [19, 20]. This extension study has been unique as a randomized multicenter prospective trial to examine the difference between statin dose-up therapy and ezetimibe add-on therapy onto baseline statin in diabetic subjects with various therapeutic backgrounds on diabetes. As in the era of multiple drugs evidenced to prevent atherosclerosis, our study would stand as a general evidence of early initiation of combination therapy. Moreover, our significant results for all the alimentary characteristics of relatively lower fat in Japanese-style meal would suggest the robust effect of the agent.

Regarding adverse events, ezetimibe add-on therapy was generally well tolerated, with an overall safety profile similar to that of a double dose of statin and consistent with previous reports [6, 21–23]. A slight but significant increase in hepatic transaminases was observed, but the patients were not subject to a clinically meaningful risk of hepatitis or persistently elevated hepatic transaminases. This is consistent with another study by adding ezetimibe [24]. Similarly, increase in creatinine level was statistically significant by double-dose statin therapy without overt episodes of renal failure. Although the effects of statins on renal diseases including diabetic nephropathy are controversial, favorable effects [25] or non-favorable effects [26] are suggested by the use of longer terms. Thus the interpretation for creatinine changes in our analysis would require longer observation.

HsCRP has been identified as a target marker of CV risk in healthy, diabetic, and secondary prevention populations. The beneficial effect of statin in reducing CV events has actually been shown to correlate not only with the statin effect on LDL-C, but also independently with the efficacy of statin in reducing hsCRP in a clinical trial using rosuvastatin [27–30]. The level of hsCRP, a significant and clinically supported target for the pleiotrophic effects of statin, was again demonstrated in the ezetimibe add-on therapy in the present trial, corroborating the former report [14]. The level of hsCRP tended to fall in both treatment arms. A significant difference from the baseline was observed at 52 weeks in both groups. The baseline hsCRP was low in the recruited diabetic subjects, but the hsCRP cut-off point for the prevention of future CHD in Japanese healthy population was only 1.0 mg/L, a value considerably lower than that reported for Western populations [31]. The ezetimibe add-on therapy actually attained a mean hsCRP of 0.5 mg/L at 52 weeks. As suggested by IMPROVE-IT study, the ezetimibe add-on therapy outperforms the statin double-dose therapy in achieving the dual target of LDL-C and hsCRP suggested by the IMPROVE-IT study [6]. Now we could conjecture here that the subjects assigned to the ezetimibe add-on therapy attained “the lower” values of these components to expect “the better” outcome more effectively than statin double dose therapy even from relatively low baseline.

Regarding the profile of atherosclerotic lipoproteins downstream of triglyceride-rich protein, the level of sd-LDLC was maintained during the 52 weeks of treatment. Dyslipidemia in subjects with type 2 diabetes or insulin resistance has been reported to be characterized by elevated concentrations of triglyceride-rich lipoproteins due increases in the synthesis of lipids, the synthesis of apo B48, or the secretion of lipoproteins by enterocytes [32–34]. In another study, type 2 diabetics expressed higher levels of NPC1L1 and MTP mRNA than control subjects [35]. These metabolic alterations lead to enriched levels of sdLDL-C and RLP-C, and subsequently to a poor prognosis of coronary artery disease associated with vulnerability of the coronary artery plaque. While the exact mechanism is unclear, our previous study in RESEARCH has shown that the inhibition of NPC1L1 protein by ezetimibe is likely to reduce the production of triglyceride-rich protein as well as the influx of cholesterol into the enterocytes [36–38]. Our extension study has demonstrated a sustainable effect of ezetimibe at the level of sd-LDL.

On the other hand, the ezetimibe add-on therapy failed to sustain the reduction of RLP-C. Fenofibrate monotherapy for type 2 diabetes has been reported to significantly decrease the concentrations of triglyceride and RLP-C, but not the concentrations of total LDL-C and sd-LDL-C [39]. Statin therapy improves the LDL particle size preferentially in subjects with insulin resistance, and high-dose statin reduces sd-LDL in non-diabetic subjects compared to a standard dose of statin [40, 41]. Tsujita et al. conducted a study to compare the co-administration of ezetimibe and atorvastatin with atorvastatin mono-therapy in Japanese patients with coronary artery disease, of whom about 30% were diabetic [7]. Treatment for 9 to 12 months resulted in a significantly lower level of LDL-C in the ezetimibe add-on therapy group even though the titration of atorvastatin targeted an LDL-C level of less than 70 mg/dl. While no differences in sd-LDL or hsCRP were found between the treatment groups in their study, the ezetimibe add-on therapy brought about a significant reduction of RLP-C. The discrepancy between their results and ours might be explained by the rate of co-morbidity with diabetes or by the lower baseline sdLDL-C level of around 32 mg/dl in their patients.

In conclusion, our study exhibited a persistently superior effect of ezetimibe add-on therapy in reducing LDL-C compared to statin dose-up therapy. Our findings were consistent with those of the IMPROVE-IT trial, which demonstrated a suppressive effect of ezetimibe on LDL-C leading to reduction in CVD events, especially in diabetics. This extension trial may support the adoption of an ezetimibe add-on strategy with a priority on achieving an LDL-C target unreachable with statins alone.

## Conclusions

In the present 52-week long-term period, ezetimibe add-on therapy showed a robust advantage in lowering LDL-C and in attaining target LDL-C values compared with the doubling of statin dose. Moreover, it’s meaningful that sd-LDL, powerfully atherogenic lipoprotein, exhibited prominent decrease consistently prominently by ezetimibe add-on therapy. DM patients with hypercholesterolemia are at high risk for CAD, and adding ezetimibe onto usual-dose statin treatment in Japan has been suggested as the first-line therapy for those DM patients who failed to attain the target LDL-C value.
